# Knowledge, Attitudes, and Coverage of Recommended Vaccinations in Individuals with Chronic Medical Conditions: A Cross-Sectional Telephone Survey in Italy

**DOI:** 10.3390/vaccines12030336

**Published:** 2024-03-20

**Authors:** Vincenza Sansone, Grazia Miraglia del Giudice, Giorgia Della Polla, Italo Francesco Angelillo

**Affiliations:** Department of Experimental Medicine, University of Campania “Luigi Vanvitelli”, Via Luciano Armanni 5, 80138 Naples, Italy

**Keywords:** attitudes, behaviors, chronic medical conditions, Italy, knowledge, patients, telephone survey, uptake, vaccinations, willingness

## Abstract

Background: This cross-sectional survey investigated the knowledge, attitudes, and coverage of recommended vaccinations among a random sample of patients with chronic medical conditions, at higher risk of vaccine-preventable diseases (VPDs), in Italy. Methods: The survey was conducted via telephone-based interviews. Results: Multinomial regression analysis showed that the patients who believed that VPDs were severe were more likely to know one recommended vaccination; those who believed that VPDs were severe and those who were advised from a general practitioner (GP) were more likely to know two vaccinations; those who were older, graduated, with more time from diagnosis, who believed that VPDs were severe, who did not need additional information, and who were advised from a GP were more likely to know three or four vaccinations. Patients who knew at least one vaccination, who perceived themselves at risk, and who were advised from a GP were more likely to have received one vaccination; those who knew more than one vaccination and who were advised from a GP were more likely to receive two or three vaccinations. Among the unvaccinated, patients who were unmarried/not cohabiting, those who needed information, and who believed that vaccinations were useful and safe were more willing to receive the recommended vaccinations. Conclusions: Educational interventions are needed to improve the adherence of individuals with chronic medical conditions.

## 1. Introduction

Patients with underlying chronic medical conditions, like cancer, diabetes, heart, liver, lung, and kidney diseases, often have a weakened immune system, making them more susceptible to certain vaccine-preventable diseases (VPDs) and their severe clinical consequences, including long-term illness, hospitalization, and even death [1,2,3,4,5]. These conditions are more likely to occur in the elderly; this is of concern since people worldwide are living longer. In Italy, according to the latest reports, the proportion of people aged 65 years and older was 24.1% and, in this context, the occurrence of multiple chronic medical conditions is more frequently accompanied by an increase in age-related diseases [6]. Indeed, 37% of those aged 65 years had at least one chronic medical condition [7]. Moreover, the invasive bacterial diseases were mainly caused by *Streptococcus pneumonia* and *Neisseria meningitidis* [8], the majority of patients admitted to hospitals because of herpes zoster (HZ) were over the age of 50 [9], and patients with chronic conditions had an increased risk for complications compared to healthy people [10].

Strategies to vaccinate those living with chronic medical conditions are very important and immunization is a public health priority. In Italy, the National Vaccination Prevention Plan actively recommends free of charge vaccinations to different categories at high-risk, including older people, pregnant women, and those with occupational exposure. In particular, specific vaccinations are recommended according to the different medical conditions, unless they are specifically contraindicated; the set target for seasonal influenza and pneumococcal among adults ≥ 65 years is 75%, for HZ among those aged ≥ 50 years is 50%, and for meningococcal ACWY among adolescents aged ≥ 15 years is 95% [11]. To vaccinate people with chronic medical conditions, the clinical care pathway must be considered at different levels, including hospital, outpatient visits, and home and territorial care with active promotion and easy access. However, a critical aspect is the patients’ adherence; there are several motivations behind the decision making to not get vaccinated, such as safety and efficacy concerns, low perceived risk of illness, lack of knowledge, and lack of information [12,13,14]. To the best of our knowledge, the number of studies in the current literature that have been conducted to assess the knowledge, attitudes, and behaviors of the recommended vaccines among individuals with chronic medical conditions in Italy are lacking [15,16,17]. Since it is important to fill the gap in the literature and considering that this understanding is crucial to increase vaccine uptake among this population, the objectives of the present cross-sectional survey were to investigate the level of knowledge, the attitudes, and the coverage with respect to recommended vaccines against seasonal influenza, pneumococcal, meningococcal, and HZ among patients with chronic medical conditions in Italy and to determine the influencing factors.

## 2. Materials and Methods

The cross-sectional telephone survey was based on the Health Belief Model which suggests that health behaviors are influenced by four types of cognitions: perceived susceptibility (perception of the risk of the health threat), perceived severity (the strength of the threat), perceived benefits, and barriers (negative consequences) of the protective/recommended behaviors.

### 2.1. Setting and Sample

The survey was approved by the Ethics Committee of the Teaching Hospital of the University of Campania “Luigi Vanvitelli”.

The survey recruited, using a simple random sampling method, over the period from July to November 2023, a total of 461 patients with a chronic medical condition, who were eligible for the vaccines against seasonal influenza, HZ, pneumococcal, and meningococcal, from the registry of those attending the Medical and Surgical Outpatient Clinics of a Teaching Hospital located in the city of Naples in the Southern part of Italy. The sample size was determined, using a single proportion formula, assuming a willingness to get the recommended vaccinations of 50%, with a margin of error of 5%, a two-sided confidence interval of 95%, and an expected 10% non-response rate. Therefore, a minimum of 427 subjects had to be interviewed.

### 2.2. Data Collection

The Health Director of the Teaching Hospital received a letter explaining the purposes and procedure of the survey and requesting the approval to administer the questionnaire. After obtaining approval from the Director, three team members, who were not affiliated with the patient’s care. contacted by mobile or home phone the potential participants from Monday to Friday between 9:00 a.m. and 8:00 p.m. in order to avoid over-sampling of non-working individuals. Two further attempts were made if no response had been received. Non-respondents were replaced with the next subsequent telephone number. The team members informed the respondents about the scope of the survey and the methodology, verified if they met the inclusion criteria for receiving at least one recommended vaccination, indicated that participation was voluntary, stated that confidentiality and anonymity of the collected information were guaranteed, and informed the participants that they could terminate their participation at any time without justification. Verbal informed consent to participate in the survey was obtained from all participants who were enrolled. Participants were not compensated for participating in this survey.

### 2.3. Questionnaire

The questionnaire was modified and adapted from previous published surveys conducted by some of us enrolling different populations [18,19]. Before the beginning of the data collection through telephone-based interviews, a pilot study was conducted on 10 participants, who were included in the final sample, to ensure feasibility, comprehensibility, and the time required to complete the questionnaire. The first section of the questionnaire collected socio-demographic and health-related characteristics, such as gender, age, partnership status, degree of education, employment, underlying chronic medical conditions, time to diagnosis, and whether they had cohabitants with chronic medical conditions. The second section investigated their knowledge of recommendations on vaccinations according with the chronic medical condition assessed by four questions with three answer options “Yes”, “No”, and “Do not know”. The third section regarded attitudes and behaviors towards infectious diseases. First, it asked about the concern of acquiring VPDs, the severity of VPDs and the usefulness of vaccinations for individuals with chronic medical conditions, with a 10-point Likert-type scale for responses, ranging from 1 = not at all to 10 = at all. In addition, two statements about the perceived risk of acquiring VPDs and the safety of vaccinations for patients with chronic medical conditions with a 5-point Likert-type scale with response options ranging from “Strongly disagree” to “Strongly agree”. Uptake of vaccinations was assessed for HZ, pneumococcal, and meningococcal with three answer options for each vaccination “Yes”, “No”, and “Do not remember”. Respondents were asked to indicate the reason/s why they received the vaccination/s and those unvaccinated to indicate whether they were willing to receive each vaccination and the underlying reasons for acceptance or refusal. Two open-ended questions were used to evaluate the number of visits with physicians in the last year and the relative reason(s). It was also asked if the general practitioner (GP) had recommended the vaccinations according with their chronic medical conditions. Moreover, the willingness to receive the seasonal influenza vaccination was investigated among those who were as yet unvaccinated. In the last section, participants were asked to indicate the sources from which they had eventually received information related to the recommended vaccinations and whether they would like to obtain further information.

### 2.4. Statistical Analysis

First, descriptive statistics, using means, ranges, and standard deviations for continuous variables, frequencies, and proportions for categorical variables, depicted the characteristics of the sample. Second, chi-square test and Student’s *t*-test were carried out to assess the predictors of the different outcomes of interest. Third, the independent variables with a *p*-value equal or less than 0.25 in the univariate analysis were incorporated into three multinomial and multivariate logistic regression models to identify which were associated with the following outcomes: knowledge that seasonal influenza, HZ, pneumococcal, and meningococcal vaccinations are recommended (none = 0; one = 1; two = 2; three or four = 3) (Model 1); having received HZ, pneumococcal, and meningococcal vaccinations (none = 0; one = 1; two or three = 2) (Model 2); and willingness to receive seasonal influenza, HZ, pneumococcal, and meningococcal vaccinations (no = 0; yes = 1) (Model 3). In Model 1, the outcome reference group was those who did not know any recommended vaccination (seasonal influenza, HZ, meningococcal, pneumococcal), compared to those who knew one, two, three or four. In Model 2, the reference group was those who did not receive any recommended vaccinations (HZ, meningococcal, pneumococcal), compared to those who have received one, two or three. The following independent variables were tested because they potentially related to all the dependent variables: gender (male = 0; female = 1), age, in years (continuous), partnership status (unmarried/separated/divorced/widowed = 0; married/cohabited with a partner = 1), baccalaureate/graduate degree (no = 0; yes = 1), number of chronic medical conditions (one = 1; two = 2; at least three = 3), time from the main chronic disease diagnosis, in years (continuous), having a cohabitant affected with chronic medical conditions (no = 0; yes = 1), concern of acquiring VPDs (continuous), considering VPDs severe for individuals with chronic medical conditions (continuous), number of visits with a physician in the last year (continuous), having received information from HCWs for the vaccinations recommended according with the chronic medical conditions (seasonal influenza, HZ, pneumococcal, meningococcal) (no = 0; yes = 1), and need of additional information on vaccines for patients with chronic medical conditions (no = 0; yes = 1). Moreover, the variables regarding believing in the usefulness of vaccinations (continuous), self-perception of the risk of acquiring VPDs (disagree/strongly disagree/uncertain = 0; agree/strongly agree = 1), and believing in the safety of vaccinations for patients with chronic medical conditions (disagree/strongly disagree/uncertain = 0; agree/strongly agree = 1) were included in Models 2 and 3; having received advice from a GP to receive the recommended vaccinations according with their chronic medical conditions (seasonal influenza, HZ, pneumococcal, meningococcal) (none = 0; one = 1; two = 2; three or four = 3) was included in Models 1 and 3; knowledge of the recommended vaccinations (HZ/pneumococcal/meningococcal) (none = 1; one = 2; two or three = 3), and having received advice from a GP to receive at least one vaccine (no = 0; yes = 1) were added in Model 2; and knowledge of recommended vaccinations (seasonal influenza, HZ, pneumococcal, meningococcal) (none = 0; one = 1; two = 2; three or four = 3) was included in Model 3. *p* = 0.2 and *p* = 0.4 were used to include or to exclude, with the stepwise selection procedure, the candidate variables from the final models. To interpret the results of the multinomial and multivariate logistic regression models, the relative risk ratio (RRR) and odds ratios (ORs), with their corresponding 95% confidence interval (CI) were used, respectively. For all analyses, two-tailed tests were performed and *p*-values ≤ 0.05 were considered statistically significant. Statistical analyses were conducted using the STATA software version 18 [20].

## 3. Results

A total of 429 subjects participated in the survey, representing 93% of the 461 patients approached. The main socio-demographic, general, and health-related characteristics of the sample are summarized in Table 1. The mean age was 52.1 years, 53.6% were males, the majority were married or cohabiting (59.6%), almost one third had a baccalaureate/graduate degree (28%), 39.6% were employed, 40.3% had at least two chronic medical conditions, and almost two thirds (63.2%) had diabetes.

### 3.1. Knowledge

One third of the sample (32.9%) did not know any vaccination recommended according with their specific chronic medical conditions, 38% were aware of one vaccination, 16.8% of two, and 12.3% of three or four. Seasonal influenza was the most recognized vaccination (63.6%), followed by pneumococcal (21.2%), HZ (18.9%), and meningococcal (10.5%). According to the chronic medical conditions, transplanted patients exhibited the highest awareness regarding seasonal influenza (70%), pneumococcal (32.5%), HZ (32.5%), and meningococcal (15%), whereas patients with cardiologic diseases indicated seasonal influenza (64.7%) and HZ (19.1%), and those with oncological diseases pneumococcal (23.8%) and meningococcal (8.3%).

Table 2 shows the results of the factors associated with the different outcomes of interest in the multinomial and multivariate logistic regression analysis. Those who believed that VPDs were severe were more likely to know one vaccination. Patients who believed that VPDs were severe, and those who had been advised from a GP to receive the recommended vaccinations according with their chronic medical conditions compared to those who had been advised for one or none were more likely to know two recommended vaccinations. Those older, those graduated, those with more time from the main chronic disease diagnosis, who believed that VPDs were severe, who did not need additional information on vaccines for patients with chronic medical conditions, and who had been advised from a GP to receive all the recommended vaccinations according with their chronic medical conditions, compared to those who had been advised for one, two, or none, were more likely to know two or three recommended vaccinations (Model 1 in Table 2).

### 3.2. Attitudes and Behaviors

Overall, regarding the attitudes, measured on a scale ranging from 1 to 10, 26.3% believed that VPDs were very severe for individuals with chronic medical conditions, with a mean value of 7.1, 15.4% self-perceived high risk to acquire VPDs, with a mean value of 5.1; 40.6% believed that the recommended vaccinations were useful for them with a mean value of 7.8. Moreover, 24.4% and 26.2% strongly agreed with the statements that they are at higher risk of acquiring VPDs because of their disease and that vaccines were safe for them, respectively.

More than half (59%) of the participants declared that they had been advised from a GP to receive vaccinations, in particular seasonal influenza was the most frequently recommended (57.8%) followed by pneumococcal (17%), HZ (10.5%), and meningococcal (7.2%). Only 16.1%, 7.2%, and 4.9% reported being vaccinated against the pneumococcal, meningococcal, and HZ, respectively. The multinomial regression analysis regarding the vaccination’s uptake indicated that those with two chronic medical conditions, those who knew one or more of the reommended vaccinations, those who perceived themselves at risk of acquiring VPDs, and those who had been advised from a GP to receive at least one of the recommended vaccinations were more likely to have received one vaccination compared with those who did not. Additionally, the patients who knew more than one vaccination and those who had been advised from a GP to receive at least one of the recommended vaccinations were more likely to receive two or three vaccinations compared with those who did not (Model 2 in Table 2).

Among those unvaccinated, only 24.5% were willing to receive the recommended vaccinations, whereas 54.6%, 38.5%, 31%, and 30% were willing to receive those against seasonal influenza, HZ, meningococcal, and pneumococcal, respectively. The multivariate logistic regression analyses showed that the patients who were unmarried/separated/divorced/widowed, those who needed additional information on vaccines for patients with chronic medical conditions, and those who believed that the vaccinations were useful and safe, were more willing to receive the recommended vaccinations (Model 3 in Table 3).

Having received a recommendation from a physician was the most frequent reason given for being vaccinated or for the willingness to receive seasonal influenza (59.3%) and pneumococcal (45.2%); however, self-protection from the disease was the most reported one for meningococcal (53.7%) and HZ (44.4%). The primary reason for the unwillingness to receive seasonal influenza and HZ was concerns about vaccination side effects (34.1% and 31.9%), whereas lack of information was the most reported one for meningococcal and pneumococcal (49.5% and 34.1%).

### 3.3. Sources of Information

Overall, 89%, 66.2%, 49.4%, and 41.3% of participants stated getting information on vaccinations against seasonal influenza, HZ, pneumococcal, and meningococcal, respectively. HCWs served as the primary source of information, mentioned by 77.6% for seasonal influenza, 57.1% for pneumococcal, 49.1% for meningococcal, and 41.2% for HZ. The second most reported source was friends/family for seasonal influenza (15.3%), pneumococcal (15.1%), and meningococcal (18.1%), whereas it was mass media for HZ (41.2%). Interestingly, half (49.5%) reported needing additional information.

## 4. Discussion

To the best of our knowledge, this current cross-sectional survey for the first time provides a comprehensive analysis that sheds light on the level of knowledge, the attitudes, and the behaviors towards the recommended vaccinations among individuals with chronic medical conditions in Italy, as well as on the potential influencing factors.

A key finding from this survey was that the level of knowledge regarding the recommended vaccinations seemed to be unsatisfactory among the surveyed patients. Notably, only less than two thirds recognized that the seasonal influenza vaccination was recommended for them (63.6%), followed by pneumococcal (21.2%), HZ (18.9%), and meningococcal (10.5%). These findings are consistent with the observations of other studies conducted in Italy among adults with chronic medical conditions and older people regarding HZ and seasonal influenza [15,21]. These gaps are worrying and could be one of the factors impeding adherence to vaccinations, which are the most effective preventive measures against diseases, especially for people with underlying chronic medical conditions with a weakened immune system who are at higher risk of severe complications from infectious diseases. Indeed, this survey identified the recommended vaccination-related knowledge as a significant predictor of patients’ behavior and willingness toward vaccinations. Respondents with adequate knowledge were more likely to have received two or three vaccinations. This aligns with evidence from previous studies which found that individuals with a higher level of knowledge were more likely to be vaccinated or to have a positive intention [15,22,23,24]. These findings underscore the critical importance of addressing knowledge gaps through targeted training and education programs since enhancing patients’ knowledge can lead to more favorable attitudes and, consequently, better participation in vaccination campaigns.

Regarding self-reported vaccination uptake, pneumococcal had the highest rate (16.1%), followed by meningococcal (7.2%), and HZ (4.9%). These findings are of extreme concern and are significantly lower than the values of 77% for seasonal influenza among Irish patients receiving immunoglobulin replacement therapy [25] and of 41% for pneumococcal vaccination among patients with chronic conditions in France [26]. Similar values of uptake have been observed for pneumococcal and influenza vaccinations with only 13.9% and 1.5% among patients with inflammatory arthritis in Bulgaria [27], 7.8% of diabetic patients in China [28], and 7% of chronic obstructive pulmonary disease patients who have received the influenza vaccine in Saudi Arabia [29]; in China, 3.29% and 6.69% of chronic disease patients aged < 65 years and ≥65 years have received the pneumococcal vaccine [30]. The multinomial logistic regression analysis showed that a higher number of comorbidities increased the likelihood of having received vaccinations, as confirmed in other studies [31,32]. One potential explanation for this association is that individuals with comorbidities may have a heightened awareness of their susceptibility to negative health outcomes. Indeed, living with multiple chronic conditions could lead to a deeper understanding of one’s health risks and frequent interactions with HCWs, providing opportunities to receive more proactive guidance and support regarding vaccination. Among unvaccinated participants, this survey found an unsatisfactory and disappointing willingness to receive the recommended vaccinations in the sample, ranging from 30% for pneumococcal to 54.6% for seasonal influenza. Higher values have been reported for seasonal influenza among elderly with chronic diseases in Tunisia (64.7%) [22] and among diabetic patients in China (46.13%) [28] and for HZ among elderly with chronic diseases in China (42.67%) [23]. These very low values are surprising also because the COVID-19 pandemic has determined in Italy a considerable number of cases and deaths among the most vulnerable individuals, like patients with chronic medical conditions. Therefore, it would have been expected that more attention would have been paid either by the patients or the HCWs toward the vaccinations with a higher uptake and recommendations although the COVID-19 pandemic has determined a negative impact on immunization activities with a decline of the coverage around the world [33,34,35].

In the current survey, a multinomial and multivariate logistic regression analysis was conducted to identify the determinants that had a significant influence on the different outcomes of interest. Specifically, the analysis revealed that the beliefs regarding VPDs and having received recommendations from GPs were predictive factors for knowledge among the sampled patients. Indeed, the level of knowledge was significantly higher among those who believed that VPDs were serious for them. This could be explained by the fact that individuals more concerned about the severity of VPDs may have a higher level of health consciousness, making them more inclined to seek information about preventive measures, including vaccinations. Another important factor influencing the knowledge was the level of education, with those that were graduated more likely to know three or four recommended vaccinations. This finding is consistent with previous surveys [15,36]. One possible explanation is that individuals with higher educational levels might have more chances to obtain information from reliable sources, which increases care for their health and well-being. Moreover, the multivariate logistic regression analyses showed that individuals who believed in the usefulness and safety of vaccinations were 1.7 and 2 times more willing to be vaccinated than those who had a negative attitude. These findings are consistent with those of previous studies conducted in different populations establishing that having positive opinions regarding vaccinations generally has a significant impact on their acceptance [37,38]. Therefore, public health education campaigns tailored to patients exhibiting skepticism towards the effectiveness and safety of vaccinations could lead to a significant increase in vaccine acceptance and uptake.

The survey showed that the participants had received vaccination-related information from HCWs, also in combination with a variety of other sources, as the most used source. This result corresponds to findings obtained in other similar surveys conducted in this and in other geographic areas [28,39,40,41,42]. Moreover, the results of the multinomial and multivariate logistic regression analysis demonstrated that HCWs were the most important communication source in disseminating accurate information about vaccinations aimed at mitigating the risk and other public health threats. Indeed, participants who had received a recommendation from HCWs were more likely to have received a vaccination. These results align with those undertaken among different populations showing that HCWs have a prominent role and the potential to influence vaccination decisions since the uptake was significantly higher among those who had had the opportunity to discuss about vaccinations with the HCWs [28,43,44,45]. However, it should be noted that only 3.3% of the respondents had been advised from their GP to receive all the recommended vaccinations; since their role is crucial in providing vaccination recommendations, it is necessary to improve the level of knowledge of HCWs and the realization of their strategic health intervention role. Furthermore, it is disturbing that only half (49.5%) of the sample expressed the need for additional information and those who had this need were more willing to be vaccinated against all the diseases they were at risk for. This finding again underscores the importance of acquiring information and this is in line with other studies which showed a significant positive role of having received information on the intention to be vaccinated [16,46]. Moreover, it should be noted that several other factors may contribute to the intention or the adherence such as, for example, the perceived risk of infection, the distrust in health authorities, and the skepticism in vaccine efficacy and safety [47,48].

Importantly, the survey provides valuable insights into the most common reasons for the respondents’ behavior and attitudes. Having received a recommendation from a physician and self-protection have been shown to be the main reasons for uptake and for the willingness to be vaccinated. By contrast, several key barriers have been identified to vaccination. The most prominent reported reason for non-vaccination or for unwillingness that was identified was misperception, like worry about safety and side-effects of the vaccines. These reasons were all or partly confirmed by the findings of several previous surveys among different populations [44,49]; up-to-date information is urgently needed to help these patients to overcome these obstacles and to improve their adherence to vaccination programs.

It is crucial to acknowledge some potential methodological limitations in interpreting the findings of this survey. Firstly, the cross-sectional survey design precluded inference of a causal relationship between the factors associated with the different outcomes of interest. Secondly, the participants were selected in a single teaching hospital and, therefore, adequate cautions might be required in the generalizability of the findings to the whole population of patients with chronic medical conditions in Italy. Thirdly, since the vaccinations uptake was assessed using a self-reported questionnaire, the quality of the data provided by the respondents was not verified by medical records, the answers may not have been highly accurate, and may have resulted in overestimation of the true coverage and misrepresentation of attitudes toward vaccinations. Fourthly, social desirability bias may occur, potentially rendering the findings less reliable since participants may tend to exaggerate their vaccination behaviors. However, this bias has been minimized by assuring the complete anonymity of participants and, therefore, one can assume that the data were reliably captured. Despite these limitations, this survey provides important enlightenment for improving the coverage of recommended vaccinations among individuals with chronic medical conditions in Italy.

## 5. Conclusions

In conclusion, this survey contributes to expanding the limited literature on the recommended vaccinations in individuals with chronic medical conditions. In light of the current results, healthcare professionals’ educational effective strategy, mainly regarding vaccine safety, efficacy, concerns, and misconceptions related to potential side effects, are needed. Counseling need to be secured to provide skills and accurate information to consistently offer vaccinations to eligible patients and to address their concerns. The findings underscore the need for efforts to enhance educational interventions and awareness by public health institutions and government policymakers to promote vaccinations and to improve the adherence of individuals with chronic medical conditions.

## Figures and Tables

**Table 1:** Socio-demographic, general, and health-related characteristics of the study population.

Characteristics	N	%
Age, years	52.1 ± 18.7 (18–91) *	
Gender		
Female	199	46.4
Male	230	53.6
Partnership status		
Married/cohabited with a partner	255	59.6
Unmarried/separated/divorced/widowed	171	40.4
Degree of education		
High school degree or less	306	72
Baccalaurate/graduate degree	119	28
Employed		
No	255	60.4
Yes	167	39.6
Number of chronic medical conditions		
One	256	59.7
Two	133	31
At least three	40	9.3
Main chronic medical conditions **		
Diabetes	271	63.2
Cancer	84	19.6
Heart disease	68	15.8
Transplantation	40	9.8
Gastrointestinal disease	27	6.3
Lung disease	11	2.6
Kidney disease	11	2.6
Time in months or years from the main chronic disease diagnosis	14.5 ± 11 (1 month-70 years) *	
Having at least one cohabitant with a chronic medical condition		
No	306	71.3
Yes	123	28.7

Number for each item may not add up to total number of the study population due to missing values. * Mean ± Standard deviation (range). ** More than one chronic medical condition was allowed to be indicated.

**Table 2:** Multinomial logistic regression analysis results with stepwise variable selection procedure examining the determinants of two outcomes of interest.

Model 1. Knowledge that seasonal influenza, herpes-zoster, pneumococcal, and meningococcal vaccinations are recommended Log likelihood = −350.73, χ^2^ = 349.42 (39 df), *p* < 0.0001									
	**One vaccination**			**Two vaccinations**			**Three or four vaccinations**		
**Variable**	**RRR**	**95% CI**	***p***	**RRR**	**95% CI**	***p***	**RRR**	**95% CI**	***p***
Considering vaccine-preventable diseases severe for individuals with chronic medical conditions	1.19	1.07–1.34	0.01	1.43	1.21–1.69	<0.001	1.41	1.17–1.69	<0.001
No need of additional information on vaccines for patients with chronic medical conditions	0.58	0.32–1.05	0.07	0.55	0.25–1.21	0.13	0.25	0.11–0.61	0.01
Younger	0.98	0.96–1.01	0.09	1.01	0.99–1.04	0.31	1.03	1.01–1.06	0.01
Having a cohabitant with a chronic medical condition	1.69	0.91–3.17	0.11	1.58	0.69–3.59	0.27	0.77	0.29–2.03	0.61
More time from the main chronic disease diagnosis	1.02	0.99–1.04	0.21	1.03	0.99–1.07	0.07	1.05	1.01–1.08	0.01
Having been advised from a general practitioner to receive the recommended vaccinations									
None	1 *								
One	0.33	0.04–2.24	0.25	0.03	0.01–0.19	<0.001	0.01	0.01–0.05	<0.001
Two	5.79	0.84–39.65	0.07	0.17	0.03–0.92	0.04	0.01	0.01–0.09	<0.001
Three or four	1.49	0.16–13.79	0.72	1.65	0.27–10.1	0.58	0.06	0.01–0.44	0.01
Married/cohabited with a partner	1.29	0.67–2.49	0.43	1.67	0.71–3.96	0.24	0.71	0.29–1.71	0.44
Fewer number of visits with a physician in the last year	0.99	0.96–1.02	0.53	0.97	0.94–1.02	0.26	0.96	0.91–1.01	0.13
Number of chronic medical conditions									
One	1 *								
Two	0.89	0.46–1.72	0.72	2.08	0.91–4.81	0.08	1.11	0.43–2.83	0.83
At least three	0.41	0.13–1.19	0.11	1.57	0.48–5.11	0.45	0.42	0.09–1.93	0.26
Graduated	1.04	0.53–2.04	0.89	2.12	0.88–5.09	0.09	2.97	1.21–7.29	0.01
Model 2. Having received herpes-zoster, pneumococcal, and meningococcal vaccinations Log likelihood = −145.26, χ2 = 225.73 (24 df), *p* < 0.0001									
	**One vaccination**			**Two or three vaccinations**					
Having been advised from a general practitioner to receive at least one recommended vaccination	12.44	5.21–29.72	<0.001	20.12	5.59–72.46	<0.001			
Knowledge of the recommended vaccinations									
None	1 *								
One	5.12	1.85–14.15	0.01	2.94	0.41–21.16	0.28			
Two or three	6.31	2.23–17.85	0.01	34.63	7.42–161.57	<0.001			
Higher perceived risk of acquiring vaccine-preventable diseases	2.55	1.11–5.84	0.02	0.77	0.25–2.41	0.66			
Number of chronic medical conditions									
One	1 *								
Two	2.42	1.04–5.62	0.04	1.43	0.45–4.57	0.54			
At least three	3.21	0.99–10.27	0.05	0.27	0.01–4.24	0.35			
Unmarried/separated/divorced/widowed	0.56	0.22–1.41	0.21	0.43	0.12–0.58	0.21			
Believing in the safety of vaccinations	1.55	0.61–4.01	0.35	1.58	0.41–6.08	0.51			
Fewer number of visits with a physician in the last year	0.98	0.93–1.03	0.43	1.03	0.97–1.08	0.29			
Older	1.01	0.98–1.03	0.53	0.97	0.94–1.01	0.17			
More time from the main chronic disease diagnosis	1.01	0.97–1.03	0.89	1.03	0.99–1.07	0.12			
Believing in the usefulness of vaccinations	1.02	0.85–1.23	0.77	1.23	0.89–1.69	0.19			

* Reference category.

**Table 3:** Multivariate logistic regression analysis results with stepwise variable selection procedure examining the determinants of the outcome of interest.

Model 3. Willingness to receive seasonal influenza, herpes-zoster, pneumococcal, and meningococcal vaccinations Log likelihood = −176.24, χ^2^ = 116.56 (10 df), *p* < 0.0001				
**Variable**	**OR**	**SE**	**95% CI**	***p***
Believing in the usefulness of vaccinations	1.66	0.18	1.34–2.05	<0.001
Need of additional information	2.71	0.78	1.53–4.78	0.01
Unmarried/separated/divorced/widowed	0.55	0.15	0.32–0.94	0.02
Believing in the safety of vaccinations	1.99	0.71	1.01–3.97	0.04
Having been advised from a general practitioner to receive the recommended vaccinations				
None	1 *			
One	1.73	0.51	0.98–3.06	0.06
Knowledge of the recommended vaccinations				
None	1 *			
One	3.56	0.17	0.31–1.02	0.06
Three or four	2.07	0.81	0.96–4.43	0.06
Having received information for the recommended vaccination from healthcare workers	1.83	0.63	0.93–3.61	0.07
Higher perceived risk of acquiring vaccine-preventable diseases	1.31	0.36	0.76–2.26	0.33
Not considering vaccine-preventable diseases severe for individuals with chronic medical conditions	0.94	0.06	0.83–1.06	0.33

* Reference category.

## Data Availability

The anonymous data presented in this survey are available on request from the corresponding author.
